# Development of programs to predict the occurrence of mucositis from digital imaging and communications in medicine data by machine learning in head and neck volumetric modulated radiotherapy

**DOI:** 10.1002/acm2.14125

**Published:** 2023-08-21

**Authors:** Toshiya Rachi, Takaki Ariji, Shinichi Takahashi

**Affiliations:** ^1^ Department of Radiological Technology National Cancer Center Hospital East Kashiwa Japan

**Keywords:** head and neck cancer, machine learning, mucositis, volumetric modulated arc therapy

## Abstract

Volumetric modulated arc therapy (VMAT) with cisplatin for head and neck cancer is often accompanied by symptoms of pharyngeal and oral mucositis. However, no standard medical program exists for the prevention and treatment of mucositis, and the mechanisms of mucositis have not yet been fully proven. Therefore, adaptive radiotherapy (ART), which is a re‐planning process, is administered when severe mucositis develops during the treatment period. We extracted the treatment plans of patients who developed severe mucositis from DICOM data and used machine learning to determine its quantitative features. This study aimed to develop a machine learning program that can predict the development of mucositis requiring ART. This study included 61 patients who received concurrent chemotherapy and radiotherapy (RT). For each patient, the equivalent square field size of each segmental irradiation field used for VMAT, dose per segment (Gy), clinical target volume high, and mean dose of the oral cavity (Gy) were calculated. Furthermore, 671 five‐dimensional lists were generated from the acquired data. Support vector machine (SVM) and K‐nearest neighbor (KNN) were used for machine learning. For the accuracy score, the test size was varied from 10% to 90%, and the random number of data extracted in each test size was further varied from 1 to 100 to calculate a mean accuracy score. The mean accuracy scores of SVM and KNN were 0.981 ± 0.020 and 0.972 ± 0.033, respectively. The presence or absence of ART for mucositis was classified with high accuracy. The classification of the five‐dimensional list was implemented with high accuracy, and a program was constructed to predict the onset of mucositis requiring ART before treatment began. This study suggests that it may support preventive measures against mucositis and the completion of RT without having to re‐plan.

## 1 INTRODUCTION

Adverse events observed with radiotherapy (RT) for head and neck cancer include oral and pharyngeal mucositis. Mucositis is particularly severe when RT is combined with chemotherapy.^1^, ^2^ Severe mucositis still occurs, and no effective preventive measures have been established. Mucositis is graded by the Common Terminology Criteria for Adverse Events v5.0 (CTCAE) guidelines.^3^ Mucositis with difficulty in oral intake is considered to be grade 3 and may affect the patient's quality of life (QOL) due to a deterioration in the nutritional status. Even in grade 2, if the patient is in severe pain that indicates a possible deterioration to grade 3, radiation therapy re‐planning is implemented. The re‐planning of the irradiation plan during the treatment period is called adaptive radiation therapy (ART).^4^ In this study, ART was adjusted to the treatment plan that has been implemented for the pharynx and oral cavity if the goal was to improve mucositis only, or a new planned computed tomography was considered if mucositis was accompanied by significant body shape changes or weight loss.

The Multinational Association of Supportive Care in Cancer and International Society of Oral Oncology (MASCC/ISOO) guidelines^5^ highlight the importance of oral care; however, this has not been proven with evidence. We considered fundamentally revising the treatment plan itself to reduce the occurrence of mucositis.

In this retrospective study, patients who underwent radical head and neck volumetric modulated arc therapy (VMAT) were differentiated, based on the presence or absence of ART. In addition, we extracted quantitative features of digital imaging and communications in medicine (DICOM) data in which mucositis had occurred, using machine learning. Factors for machine learning classification and the amount of change in the target during treatment have been investigated in previous studies.^6^, ^7^ We aimed to develop a program that can predict the occurrence of mucositis by reading the DICOM data and the treatment plan. If the classification accuracy of the ART implementation group was high, it could predict the development of severe mucositis requiring ART.

## 2 METHODS

### 2.1 Target patients and treatment plans

**FIGURE 1:**
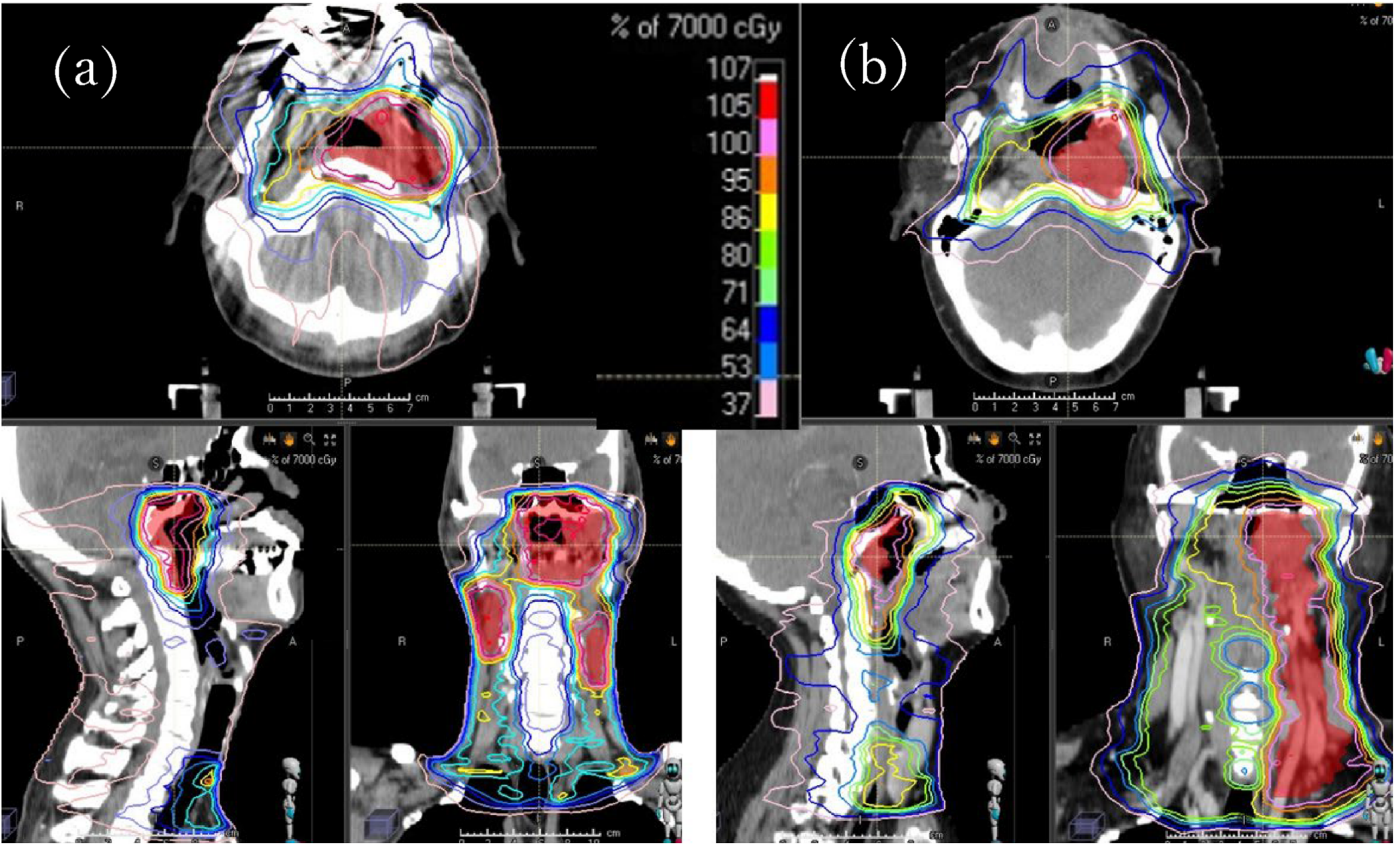
Dose distribution for cases that did not develop mucositis during treatment (a) and for cases that developed grade 3 mucositis (b).

A total of 61 patients were included in the study: 14 patients with nasopharyngeal cancer, 28 with oropharyngeal cancer, 14 with hypopharyngeal cancer, and five with tongue cancer. All patients underwent concurrent chemoradiotherapy with cisplatin, and VMAT was used as the irradiation technique. These patients were divided into two groups: 43 who were able to complete radiation therapy without ART in the ART‐naive group, and 18 with grade 2 or more mucositis requiring ART. Patients receiving ART owing to mucositis are those who develop mucositis during the course of treatment that affects their QOL and for whom the radiation oncologist determines that they cannot complete radiation therapy without a change in treatment plan. ART was administered once to 18 patients during the treatment period, and all patients completed RT at the planned dose. Figure 1 shows a comparison of dose distributions for cases that did not develop mucositis during treatment (a) and those that developed grade 3 mucositis (b).RayStation ver 10A (RaySearch Medical Laboratories AB, Stockholm, Sweden) was used as the treatment‐planning device. One dose fraction was 2 Gy, with prescribed doses of 66−70 Gy for high‐risk clicical target volume (CTV), 60−63 Gy for intermediate‐risk CTV, and 54−56 Gy for low‐risk CTV.^8^, ^9^ Newly irradiated target regions for ART, which is the re‐planning strategy, reduced planning target volume margins in the pharyngeal mucosa and perioral area according to the radiation oncologist. No major changes were made to the optimization parameters of the treatment plan, and the multileaf collimator (MLC) was fitted to the modified target by repeating the optimization calculations.

The linear accelerators (LINACs) used were TrueBeam and Clinac iX (Varian Medical Systems, Palo Alto, CA). Krishnappan et al. reported that the two LINACs were within clinically acceptable limits, as the variation in the dose difference between the two devices was <3%.^10^ The beam angle used was 1 or 2 arcs with full rotation irradiation. Segments were calculated at every 4°, resulting in 90 segments of treatment with the use of 1 arc and 180 segments with the use of 2 arcs with 360° rotation.

### 2.2 Data extraction for machine learning from RT plan and RT structure

The machine learning dataset extracted from each patient data included the monitor unit (MU) of each beam, location of the MLC in each segment of those beams, and dose irradiated in each segment (Gy) were obtained. Furthermore, the area of the irregularly shaped irradiation field for each segment was calculated from the position and thickness of the MLC, and the equivalent square irradiated field (EQIF) sizes for the same area were calculated. The EQIF sizes were divided into 11 ranges of 3, 4, 5, 6, 8, 10, 12, 14, 16, 18, and 20 cm^2^. Moreover, the irradiated dose for each segment was accumulated to the corresponding EQIF size. This accumulated dose to the EQIF was repeated for the number of target patients to determine the difference in the segmental irradiation field area used for the ART group due to mucositis and the non‐ART group.

Next, CTV high, CTV intermediate and CTV low contoured by the radiation oncologist were divided based on the body mass index (BMI) of each patient and calculated as CTV per unit BMI (CTV/BMI). CTV high, CTV intermediate, and CTV low contoured by the radiation oncologist on the initial planning computed tomography, were divided based on the BMI of each patient, and calculated as CTV per unit BMI (CTV/BMI). This compensated for differences in CTV due to differences in body size. The *t*‐ and *z*‐tests were performed with 5% precision to determine whether there was a difference in CTV/BMI between the ART and non‐ART groups in the initial planned CT contouring. This determined if the CTV of the ART group was larger than that of the non‐ART group.

In addition, the mean dose administered in the oral cavity was obtained from the dose‐volume histogram. This may be a significant factor for ART implementation in mucositis symptoms, which has been reported by Moslemi et al.^1^

A five‐dimensional list of EQIF size (cm^2^), CTV high/BMI (cm^3^▪m^2^/kg), total MU, irradiation dose (Gy) for each segment, and mean dose administered to the oral cavity was generated for each patient. The reason for selecting CTV high/BMI is described in the Results section below. For each patient, the EQIF size (cm^2^) and corresponding segmental dose (Gy) were 11 data each, and CTV high/BMI (cm^3^‐m2/kg), total MU, and the average oral dose were one data each; hence, the number of five‐dimensional lists was 11 per patient. Since there were 61 patients, a total of 671 five‐dimensional lists were generated, and these lists were divided into two groups based on whether ART or non‐ART was administered.

### 2.3 Classification by program environment and machine learning

**FIGURE 2:**
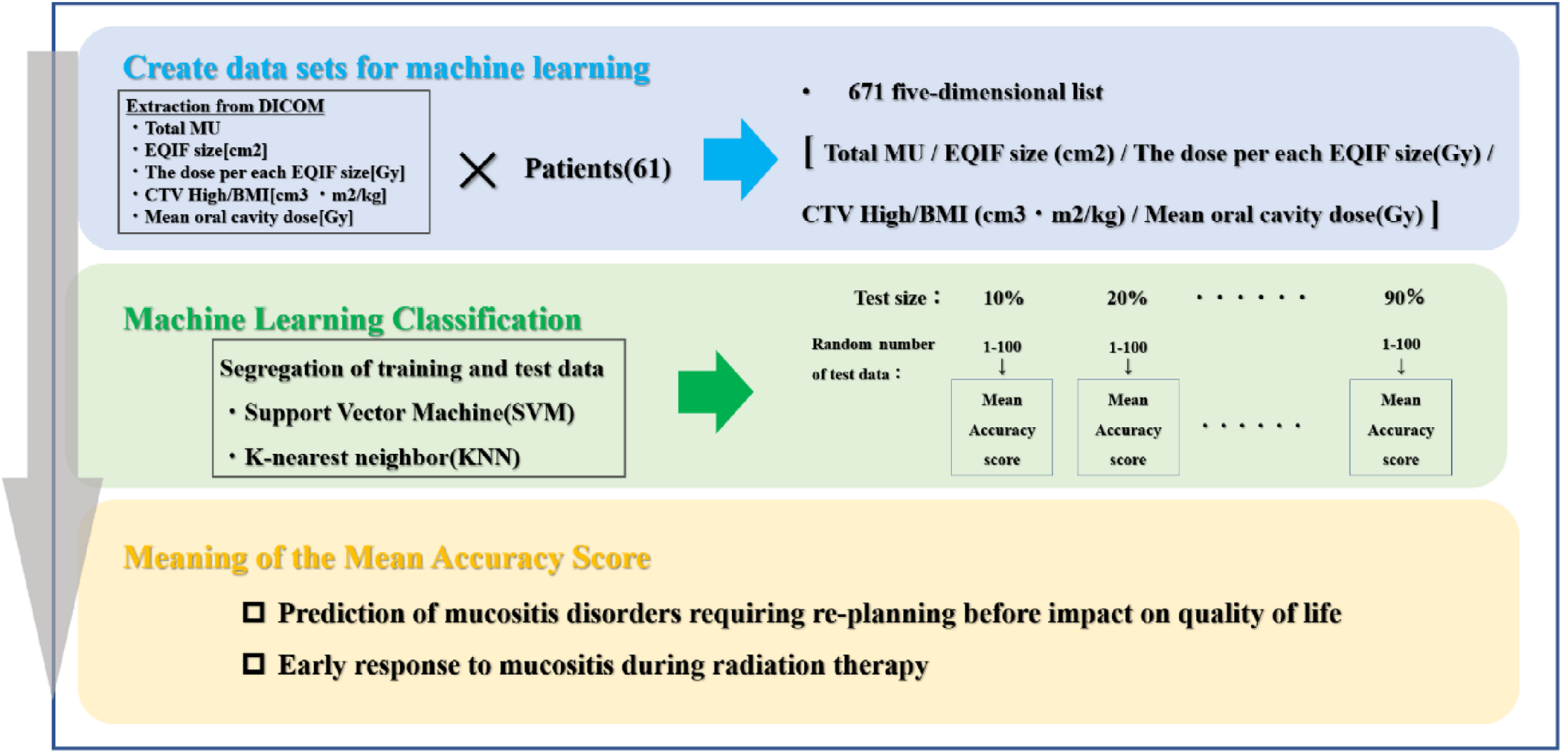
The flowchart shows the process from data set extraction, classification by machine learning with SVM and KNN, and its accuracy verification. CTV, clinical target volume; EQIF, equivalent square irradiated field; KNN, K‐nearest neighbor; MU, monitor unit; SVM, Support vector machine.

Python 3.7.6 was used to develop two machine learning programs and RT plan was loaded using the pydicom library.^11^ Two programs were implemented to classify the presence or absence of ART implementation for the five‐dimensional list created. These programs utilized a Support Vector Machine (SVM) classifier based on the Gaussian kernel (Radial Basis Function: RBF),^12^, ^13^ and a K‐nearest neighbor (KNN) classifier.^14^ SVM is versatile and capable of high‐dimensional classification. The algorithm of the KNN method is simple and user‐friendly, and its prediction results are not black‐boxed; therefore, we hypothesized that comparing the results of SVM with those of KNN will increase the reliability of SVM. Figure 2 shows the flow from splitting the learning and test data to training and accuracy score calculation. The following equation provided the dataset to the SVM:

(1) $$\left(x_{1},y_{1}\right),\text{…},\left(x_{n},y_{n}\right),x_{i}\in R^{d}\;\mathrm{and}\;y_{i}\in \left(-1,+1\right)$$

*x_i_* denotes the feature vector of the five‐dimensional lists, and *y_i_* denotes the class label. The optimal hyperplane was defined as follows:

(2) $$wx^{T}+b=0$$

*w* is the weight vector, *x* is the input feature vector, and *b* is the bias; *w* and *b* satisfy the following inequality for all elements:

(3) $$wx_{i}^{T}+b\geq +1\;\mathrm{if}\;y_{i}=1$$

(4) $$wx_{i}^{T}+b\leq +1\;\mathrm{if}\;y_{i}=-1$$

In SVM, the optimal hyperplane separates the data and estimates *w* and *b* that maximize the margin *1/||w||2*. KNN is a simple method that classifies the features of the input values into a large group belonging to the n data with the closest Euclidean distance, as shown below:

(5) $$d{\left(p,q\right)}^{2}={\left(q_{1}-p_{1}\right)}^{2}+{\left(q_{2}-p_{2}\right)}^{2}$$

*p* and *q* are the coordinates of the two points, calculated as the positive square root of the sum of the squares of the differences in each axis.

First, the 671 five‐dimensional lists were split into learning and test data in arbitrary proportions. The test size varied from 10% to 90% at 10% intervals. The random number related to the extracted test data varied from 1 to 100 at each test size, and the mean accuracy score was calculated. The SVM parameters were set to gamma = 0.05, *c* = 10, and KNN parameters were set to *n* = 5. Owing to the risk of bias that may arise from insufficient data or over‐learning, size of data to be extracted, and random number value, we varied the data size and randomness to ensure that the results were not biased or misleading. The risk of bias increases the likelihood that features of the study design or conduct of the study will give misleading results.

## 3 RESULTS

### 3.1 Comparison of CTV high, CTV intermediate, and CTV low with and without ART

**FIGURE 3:**
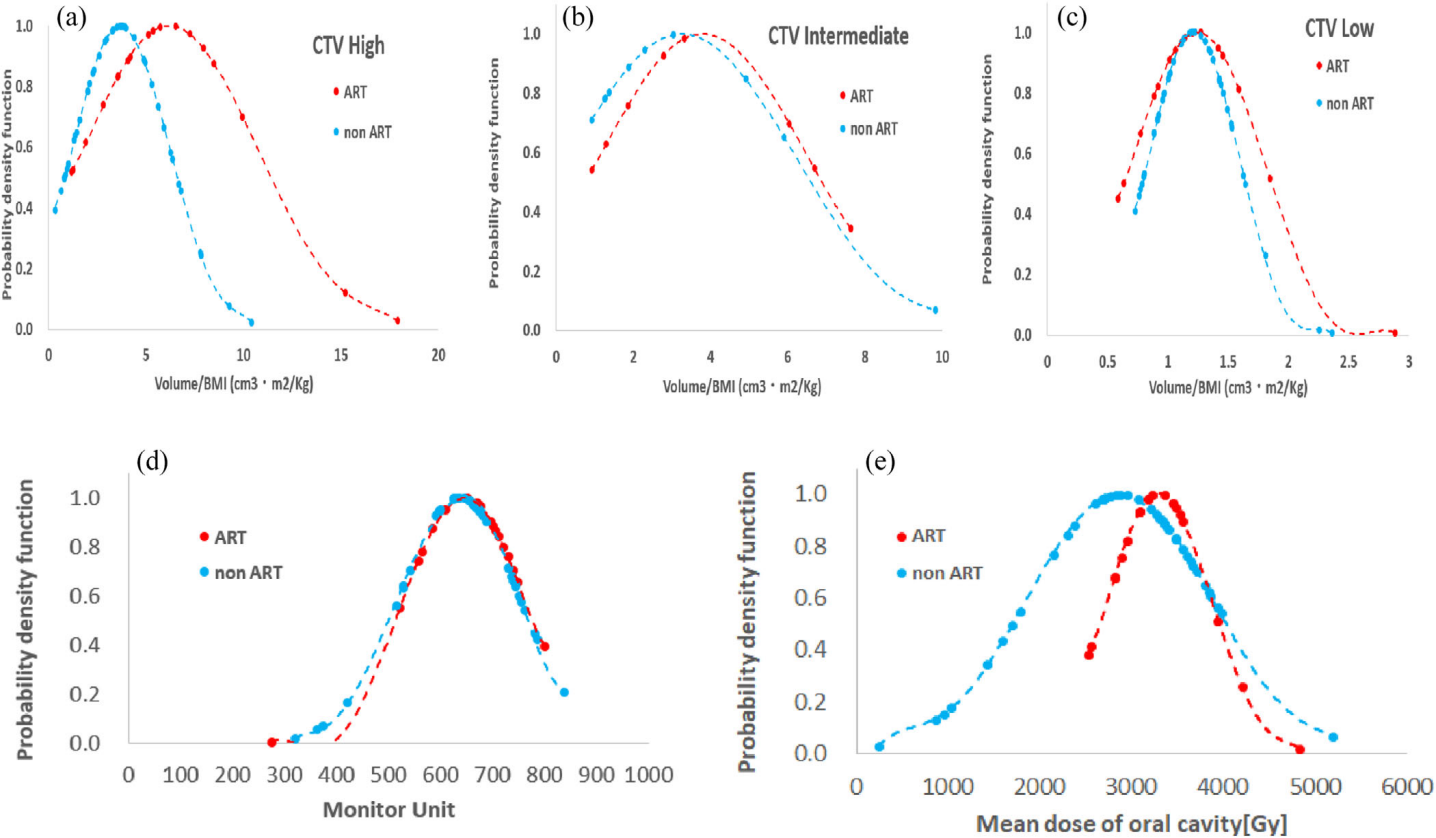
The probability density distribution graph of the volume per unit BMI of CTV high, CTV intermediate, and CTV low, and the probability density distribution graph of the total MU are shown here. In the upper three figures, the horizontal axis represents the CTV high/BMI (a), CTV intermediate/BMI (b), and CTV low/BMI (c), and the vertical axis represents the probability density. The red color is the ART group and the blue color is the non‐ART group. The largest difference between the red and blue curves is observed in the CTV high‐risk areas. The figure on the lower sides shows the probability density function of the total MU (d) and the mean oral cavity dose (e) of the ART and non‐ART groups. No significant difference was observed for MU in the *z*‐ and *t*‐tests; however, it was observed that the probability density of the ART group (red) was slightly higher than that of the non‐ART group (blue). A clear divergence was observed in the mean oral dose with and without ART. ART, adaptive radiotherapy; BMI, body mass index; CTV, clinical target volume; MU, monitor unit.

Figure 3 shows the comparison for each risk volume divided by the presence or absence of ART implementation. It represents the probability density function on the vertical axis and CTV per unit BMI (cm^3^▪cm^2^/kg) on the horizontal axis. Table 1 presents the results of these tests, with the largest difference observed between the red and blue curves in the CTV high areas. The *p*‐values of the *t*‐test for the CTV high, CTV intermediate, and CTV low were 0.036, 0.676, and 0.812, respectively, and that of the *z*‐test were 0.026, 0.671, and 0.810, respectively. The smallest *p*‐value was calculated for CTV high in both tests, and significant differences were observed in both the *t*‐ and *z*‐tests (*p* < 0.05). Based on these results, the CTV high/BMI with the largest difference was selected for the five‐dimensional list.

**TABLE 1:** Mean values of CTV/BMI, total MU and oral cavity dose with/without ART for mucositis, and *p*‐values of the *t*‐test and *z*‐test, respectively.

	*Mean: Non‐ART*	*Mean: ART*	*p‐value (t‐test)*	*p‐value (z‐test)*
**CTV High**	**3.70**	**6.21**	**0.036**	**0.026**
**CTV Intermediate**	**3.26**	**3.81**	**0.676**	**0.671**
**CTV Low**	**1.21**	**1.24**	**0.812**	**0.810**
**ＭＵ**	**636**	**644**	**0.801**	**0.814**
**Mean oral cavity dose**	**2870**	**3298**	**0.040**	**0.035**

*Note*: Significant differences were observed in CTV high and mean oral cavity dose (*p* < 0.05).

Abbreviations: ART, adaptive radiotherapy; BMI, body mass index; CTV, clinical target volume; MU, monitor unit.

### 3.2 Comparison of total MU and mean dose administered to the oral cavity, and the integrated dose used for each EQIF size with and without ART

**FIGURE 4:**
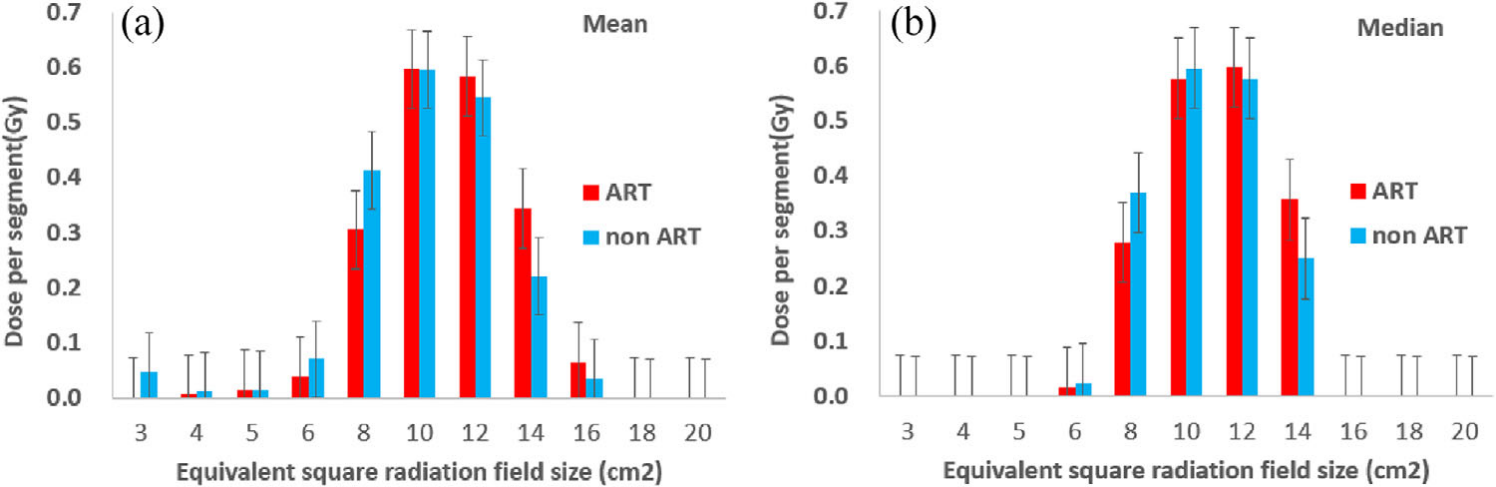
The mean and median integrated doses by EQIF size. Both the mean (a) and median (b) doses in the ART group was higher for EQIF sizes larger than 12 × 12 cm^2^, indicating that higher doses were used in a larger irradiation field. ART, adaptive radiotherapy; ART, adaptive radiotherapy; EQIF, equivalent square irradiated field.

The difference in total MU and mean dose administered in the oral cavity between the ART and non‐ART groups was tested in the same way as CTV, and the results are shown in Figure 3 and Table 1. The *p*‐values of the MU *t*‐ and *z*‐tests were 0.801 and 0.814, respectively, and no significant difference was observed (*p* > 0.05). The mean dose administered in the oral cavity was 2870 and 3298 cGy for the non‐ART and ART groups, respectively, and the *p*‐values were significantly different for both the *t*‐ and *z*‐tests at 0.040 and 0.035, respectively. A large discrepancy was observed between the ART and non‐ART groups as shown in Figure 3. Table 2 shows the average and median integrated doses for each EQIF size. For the 10 × 10 cm^2^ and 12 × 12 cm^2^ EQIF sizes, the average and median dose difference between the ART and non‐ART groups were less than +7% relative to the non‐ART group. Furthermore, for the 6 × 6 cm^2^ and 8 × 8 cm^2^ EQIF sizes, the non‐ART group had higher doses, with a dose difference > 20% for both average and median. The *p*‐values were both low, with a significant difference observed at the 8 × 8 cm^2^ EQIF size (*p* < 0.05). Conversely, for the 14 × 14 cm^2^ and 16 × 16 cm^2^ EQIF sizes, the average and median doses were +56.16% and +42.59%, respectively, showing a large difference with higher doses in the ART group. The *p*‐values were also lower in the wider EQIF, and a significant difference was observed for the 14 × 14 cm^2^ size (*p* < 0.05). The ART group used higher doses in the EQIF size of 12 × 12 cm^2^ or larger for both the mean and median values, indicating that more irradiation dose was used in a larger irradiation field. This was also observed in Figure 4, where the red ART and blue non‐ART doses were reversed for the 12 × 12 cm^2^ size.

**TABLE 2:** Average and median EQIF size (cm^2^) calculated from each segment per patient.

	Average			Median			
EQIF size (cm^2^)	ART	Non‐ART	Relative difference (%)	ART	Non‐ART	Relative difference (%)	*p‐value*
**3**	**0**	**0.0469**	**−100**	**0**	**0**	**–**	**0.29**
**4**	**0.0061**	**0.0113**	**−45.99**	**0**	**0**	**–**	**0.60**
**5**	**0.0140**	**0.0142**	**−1.35**	**0**	**0**	**–**	**0.99**
**6**	**0.0382**	**0.0703**	**−45.62**	**0.0155**	**0.0237**	**−34.57**	**0.10**
**8**	**0.3051**	**0.4138**	**−26.28**	**0.2798**	**0.3698**	**−24.35**	**0.04**
**10**	**0.5972**	**0.5957**	**0.24**	**0.5768**	**0.5960**	**−3.22**	**0.97**
**12**	**0.5844**	**0.5449**	**7.25**	**0.5979**	**0.5775**	**3.52**	**0.37**
**14**	**0.3440**	**0.2202**	**56.16**	**0.3572**	**0.2505**	**42.59**	**0.03**
**16**	**0.0653**	**0.0349**	**86.88**	**0**	**0**	**–**	**0.29**
**18**	**0**	**0**	**–**	**0**	**0**	**–**	**–**
**20**	**0**	**0**	**–**	**0**	**0**	**–**	**–**

*Note*: Each segment was differentiated by ART implementation, with the high side shown in red and the low side in blue. For 12 × 12 cm^2^ and above, both the average and median were higher in the ART group than those in the non‐ART group.

Abbreviations: ART, adaptive radiotherapy; EQIF, equivalent square irradiated field.

### 3.3 Classification of the five‐dimensional lists by machine learning

The results of the classification of 671 five‐dimensional lists are summarized in Table 3. When the test size was between 0.1 and 0.2, the SVM and KNN obtained the highest accuracy value of 0.999 and 1.000, respectively. The lowest values were obtained when the test size was 0.9 (90% of test data, 10% of learning data), with accuracy scores of 0.937 and 0.905 for SVM and KNN, respectively. Both SVM and KNN showed high values, with a mean ± standard deviation of 0.981 ± 0.020 and 0.972 ± 0.033, respectively, and the coefficient of variation was 0.021 and 0.034, respectively. The low coefficient of variation was independent of the extracted test data and test size, and was directly related to the small inter‐individual differences in CTV contouring and optimization parameters required for CTV treatment planning.

**TABLE 3:** SVM and KNN mean accuracy scores with the mean values of random numbers (1−100) for each test size.

SVM	Test size											
	0.1	0.2	0.3	0.4	0.5	0.6	0.7	0.8	0.9	Mean	SD	CV
**Mean value (Random number, 1−100)**	**0.999**	**0.999**	**0.997**	**0.994**	**0.990**	**0.982**	**0.972**	**0.957**	**0.937**	**0.981**	**0.020**	**0.021**

KNN	**Test size**											
	**0.1**	**0.2**	**0.3**	**0.4**	**0.5**	**0.6**	**0.7**	**0.8**	**0.9**	**Mean**	**SD**	**CV**
**Mean value (Random number, 1−100)**	**1.000**	**1.000**	**0.999**	**0.996**	**0.989**	**0.976**	**0.955**	**0.930**	**0.905**	**0.972**	**0.033**	**0.034**

Abbreviations: KNN, K‐nearest neighbor; SVM, Support vector machine.

Although no significant differences were observed among all of the items in the five‐dimensional lists, which consisted of information such as patient information and dose distribution before treatment, the SVM and KNN generated in this study were able to classify the presence or absence of ART administered for mucositis with approximately 100% accuracy.

## 4 DISCUSSION

The acute‐phase effects of RT for head and neck cancer include xerostomia, taste disorders, and pharyngeal and oral mucositis.^7^ Among these, mucositis is almost inevitable regardless of the severity of the disease, and grade 3 or higher can affect the QOL, completion of radiation therapy, and prognosis.^15^ The peri‐operative goals during RT are twofold: (a) prevention of secondary infection and (b) reversal of the mucositis that has occurred. Although the use of antiseptics and steroid ointments as anti‐inflammatories, which have been shown to be effective in preventing infection,^1^ is desirable, there is little evidence that they are effective in preventing severe infections. The lack of the prevention of, or response to, mucositis highlighted in various reviews has been reported by Moslemi et al.^1^ It is important to predict and prevent the occurrence of mucositis requiring re‐planning from patient information and treatment planning data, as in this study. No report similar to this study has been found worldwide. While using VMAT for treating head and neck cancers, identifying the characteristics of treatment plans with mucositis requiring re‐planning from DICOM data and feed back to the treatment plan to support completion of radiation therapy without re‐planning is necessary.

Pharyngeal and oral mucositis is caused by radiation and chemotherapy, as well as by induced cytokines and free radicals that damage the mucosal epithelial basal cells and induce apoptosis.^16^ Concurrent chemoradiotherapy with cisplatin for head and neck cancer has been reported to be significantly more toxic than radiation therapy alone.^17^ In general, mucositis is expected to be mild at doses ≤30 Gy, with an increased likelihood of suppuration and bleeding at doses > 30 Gy.^18^ Allison et al. reported that in head and neck RT, patients who received irradiation of the mid‐pharynx with a mean dose exceeding 30 Gy had a higher need of parenteral nutrition.^19^ Narayan et al. also found that the severity of mucositis was milder (grade ≤ 1) and shorter in duration (≤1 week) when the cumulative point dose was < 32 Gy.^20^ Figure 1 shows that in the dose distribution of ART patients, the blue isodose curve that produces mucositis extends slightly more toward the oral cavity than that of non‐ART patients. The number of irradiations for the patients in this study was 33−35 fractions, and dividing 30 Gy by the number of fractions used yields 0.85−0.9 Gy per fraction. The doses used to reach these thresholds are listed in Table 2, with and without ART, with a larger contribution from doses irradiated in EQIF sizes ranging from 8 × 8 cm^2^ to 14 × 14 cm^2^.

Furthermore, significant differences were only observed in CTV high/BMI, and the use of higher doses and wider irradiation fields may be associated with a greater CTV high/BMI in the ART group than that in the non‐ART group. Therefore, the selection of CTV high/BMI for the five‐dimensional lists was considered optimal when considering mucositis requiring ART. In Table 2, no significant differences are observed between the ART and non‐ART groups for all EQIF sizes from 8 × 8 cm^2^ to 14 × 14 cm^2^. However, the dose is higher in the non‐ART group for the 6 × 6 cm^2^ and 8 × 8 cm^2^ EQIF sizes, and the ART group for the 14 × 14 cm^2^ and 16 × 16 cm^2^ EQIF sizes. The reason for this may be considered to be that the CTV High/BMI of the ART patients is greater than that of the non‐ART patients. Perhaps treatment planning could be adjusted to use fractionated irradiation fields with equivalent square field sizes of 12 × 12 cm^2^ or smaller, which could reduce mucositis. Yoosuf et al. varied the minimum interleaf width of the MLC in VMAT treatment planning and evaluated the created treatment plans using the dose‐volume histogram.^21^ They found that it is possible to limit the segmental irradiation field used by adjusting the parameters of the MLC when creating VMAT.

On the other hand, SVM^22^ and KNN^23^ are known to guarantee high classification accuracy even at high dimensions, and the five‐dimensional list was an appropriate target. Among them, the mean oral dose was the most important classification factor. As noted in Table 1, the average oral dose for the ART group was 3298 cGy, which was also higher than the dose (30–32 Gy) suggested by Allison et al.^19^ and Narayan et al.,^20^ and the non‐ART group was 2870 cGy, which was lower than that. This was consistent with their findings. In addition, Wang et al. showed that only 25% of patients in the spare group with a mean oral dose ≤3200 cGy had grade 2 mucositis or higher, whereas 100% of the patients in the no‐spare group had grade 2 or higher.^24^ No significant differences were observed in each of the terms included in the five‐dimensional list; however, we considered that the small differences that occurred in each, and the difference in the mean oral cavity dose, which allowed for the highly accurate classification of ART implementation.

The limitation of this study was that it was a non‐randomized trial that predicted the occurrence of severe mucositis in patients who required ART. Furthermore, CTV may change over the course of treatment, and BMI and mean intraoral dose are only available at a certain point in time (before treatment). Other limitations such as changes in BMI and dose as treatment progresses were not considered. In the future, periodic CTV drawings, BMI, and average intraoral dose should be collected to make the data more robust.

However, classification by SVM and KNN against a five‐dimensional list generated from pre‐treatment information was highly accurate and was considered a quantitative indicator sufficient to suspect the possibility of ART due to mucositis. Since this study used a general‐purpose program, it can be conducted at any facility. We believe that we have proposed an innovative method using a novel machine learning technique.

## 5 CONCLUSION

In this study, the five‐dimensional lists consisting of irradiation parameters extracted from the RT plan, which is DICOM data, including the CTV high, which is included in RT structure, total MU, EQIF size, irradiation dose, and mean oral dose, were generated. In addition, a program for predicting the occurrence of mucositis damage was developed using the SVM and KNN classifiers, which are machine learning methods, with supervised data. The classification of the five‐dimensional lists by these programs was implemented with high accuracy and was indicated to be able to predict the onset of severe mucositis requiring ART before the start of treatment. The results of this study are meaningful to clinicians in terms of treatment planning and patient care, and also beneficial for improving the QOL of patients.

## AUTHOR CONTRIBUTIONS

The following is my response to your request. Takaki Ariji gave me advice on the accuracy of the text and against mucositis; Shinichi Takahashi analyzed all the data and created the tables; I am now working on the data for the next few months; I am working on the data for the next few months. Toshiya Rachi, the author, analyzed all data and built the program.

## CONFLICT OF INTEREST STATEMENT

The authors declare no conflicts of interest.

## ETHICS STATEMENT

This study is a retrospective study of RT. Ethical approval for the use of existing patient data has been obtained from the Ethics Committee of the National Cancer Center Hospital in Japan (Data 12 Oct 2020/No.2020‐272).

## Data Availability

Research data are stored in an institutional repository and will be shared upon request to the corresponding author.
